# Methionine: An Indispensable Amino Acid in Cellular Metabolism and Health of Atlantic Salmon

**DOI:** 10.1155/2023/5706177

**Published:** 2023-10-27

**Authors:** M. Espe, A. C. Adam, T. Saito, K. H. Skjærven

**Affiliations:** Institute of Marine Research, P.O. Box 5817 Nordnes, Bergen, Norway

## Abstract

Methionine is an indispensable amino acid with an important role as the main methyl donor in cellular metabolism for both fish and mammals. Metabolization of methionine to the methyl donor S-adenosylmethionine (SAM) has consequence for polyamine, carnitine, phospholipid, and creatine synthesis as well as epigenetic modifications such as DNA- and histone tail methylation. Methionine can also be converted to cysteine and contributes as a precursor for taurine and glutathione synthesis. Moreover, methionine is the start codon for every protein being synthetized and thereby serves an important role in initiating translation. Modern salmon feed is dominated by plant ingredients containing less taurine, carnitine, and creatine than animal-based ingredients. This shift results in competition for SAM due to an increasing need to endogenously synthesize associated metabolites. The availability of methionine has profound implications for various metabolic pathways including allosteric regulation. This necessitates a higher nutritional need to meet the requirement as a methyl donor, surpassing the quantities for protein synthesis and growth. This comprehensive review provides an overview of the key metabolic pathways in which methionine plays a central role as methyl donor and unfolds the implications for methylation capacity, metabolism, and overall health particularly emphasizing the development of fatty liver, oxidation, and inflammation when methionine abundance is insufficient focusing on nutrition for Atlantic salmon (*Salmo salar*).

## 1. Introduction

**Figure 1:**
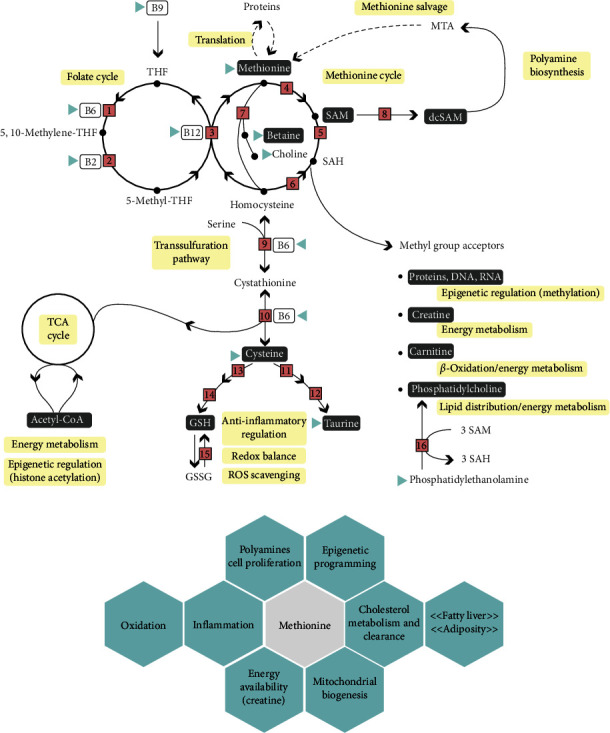
Methionine and one-carbon (1C) cycle—core to metabolism and multiple biological functions with importance for health and disease states. (a) The indispensable amino acid methionine represents the initiating amino acid in protein translation and functions as methyl donor for a range of metabolites through its conversion to SAM. Methionine also intervenes in energy metabolism, lipid storage, and transport, and counteracts oxidative stress through transsulfuration to produce glutathione (GSH). Decarboxylation of SAM (dcSAM) is necessary for polyamine synthesis and methionine is regenerated through the salvage pathway. Metabolization and recycling of methionine depend on the availability of certain macro- and micronutrients, particularly B-vitamins (B6, B9, and B12) functioning as cofactors or substrates in folate and methionine cycle. (b) Dietary methionine and its metabolization play a crucial role for both healthy metabolism and disease states as its availability affects diverse biological functions. Dark gray rectangles: metabolites or derivates of methionine emphasized in this review. Yellow rectangles: biological functions or metabolic pathways. Blue–green triangles: dietary source or composition (if natural or supplemented) can impact the availability of a nutrient or metabolite. Numbered enzymes: 1: serine hydroxymethyltransferase 1, 2 : 5, 10-methylenetetrahydrofolate reductase, 3: methionine synthase, 4: S-adenosyl methyltransferase, 5: glycine N-methyltransferase, 6: S-adenosyl-L-homocysteine hydrolase, 7: betaine-homocysteine methyltransferase, 8: SAM decarboxylase, 9: cystathionine *β*-synthase, 10: cystathionine *γ*-lyase, 11: cysteine dioxygenase, 12: cysteine sulfinate decarboxylase, 13: glutamate–cysteine synthase, 14: glutathione synthase, 15: NADPH-dependent GSH reductase, and 16: phosphatidylethanolamine transferase. Abbreviations not explained in the review: DNA: deoxyribonucleic acid, GSSG: glutathione disulfide, MTA: 5′-methylthioadenosine, RNA: ribonucleic acid, THF: tetrahydrofolate.

Methionine is among the indispensable and sulfur-containing amino acids. It becomes a limiting amino acid in modern feeds for Atlantic salmon as they contain more plant protein sources than fish meal or other marine sources like krill. Methionine plays a vital role in promoting fish growth, but its abundancy is also critical for the synthesis of other metabolites that depend on its availability. For example, taurine, carnitine, and creatine are abundant in animal-derived ingredients, and a reduction in their inclusion necessitates a higher reliance on methionine. Cysteine, another sulfur-containing amino acid, is considered conditionally indispensable as it can be synthetized from methionine (Figure 1(a)) but not vice versa. Both methionine and cysteine are incorporated into proteins. When estimating the nutritional requirement for methionine, it is crucial to consider both the dietary incorporated amount of methionine and cysteine into the calculations.

The methionine requirement to retain good health status, as indicated by low-relative liver weight, in adult salmon is 7 g methionine/kg diet when fed 6 g cysteine/kg diet [1]. In adult salmon, neither growth nor protein accretion was affected by methionine delivery, but in juvenile salmon, a diet containing 7.9 g methionine/kg and 5.7 g cysteine/kg diet reduced growth due to a decrease in protein gain compared to fish fed a similar diet containing 11.4 g methionine/kg diet indicating a higher requirement for juveniles compared to adult salmon [2]. Recently, Wang et al. [3] reviewed methionine requirement in several fish species based on weight gain and feed efficiency. Most often the determination of methionine requirement is based on weight gain and/or feed efficiency, but methionine requirement varies for growth and metabolism. Considering that dietary methionine levels also influence levels of the main methyl donor S-adenosylmethionine (SAM), it is not surprising that dietary methionine not only affects the growth but also metabolism and overall health status (Figure 1(b)). This review provides a comprehensive summary of the existing knowledge regarding the metabolic significance of methionine and its conversion into diverse metabolites under varying conditions with consequences for methylation capacity, metabolism, and overall health encompassing aspects such as development of fatty liver, oxidation, and inflammation focusing on Atlantic salmon.

## 2. Methionine and 1C Metabolism

**Figure 2:**
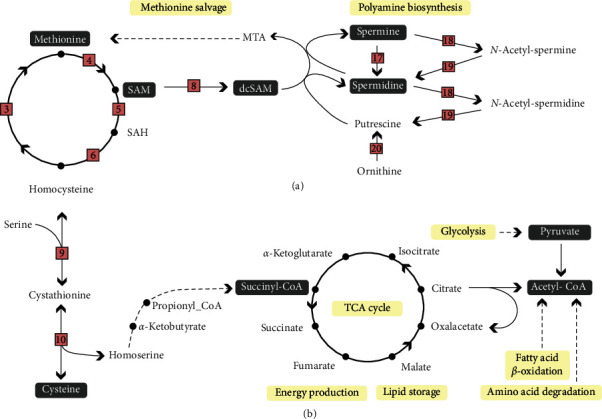
Methionine abundance determines polyamine synthesis and TCA cycle. (a) The polyamines spermidine and spermine are synthetized from putrescine (from ornithine) and decarboxylated S-adenosylmethionine (dcSAM), which is synthetized from methionine through decarboxylation of SAM. Polyamine concentrations are regulated by acetylation to lower polyamines or by being oxidized. Methionine gets regenerated from 5′-methylthioadenosine (MTA) through the methionine salvage pathway. (b) Excess methionine is eliminated through transsulfuration to cysteine or through entering the tricarboxylic acid (TCA) cycle as succinyl-CoA and thereby intervening in both energy metabolism and lipid storage. Acetyl-CoA results from amino acid degradation, glycolysis through pyruvate or fatty acid *β*-oxidation, and fuels the TCA cycle. Dark grey rectangles: metabolites or derivates of methionine emphasized in this review. Yellow rectangles: biological functions or metabolic pathways. Numbered enzymes: 3: methionine synthase, 4: S-adenosyl methyltransferase, 5: glycine N-methyltransferase, 6: S-adenosyl-L-homocysteine hydrolase, 8: SAM decarboxylase, 9: cystathionine *β*-synthase, 10: cystathionine *γ*-lyase, 17: spermidine oxidase, 18: spermidine/spermine-N1-acetyltransferase, 19: acetylated polyamine oxidase, and 20: ornithine decarboxylase. Abbreviation not explained in the review: MTA: 5′-methylthioadenosine, THF: tetrahydrofolate.

Methionine is involved in several metabolic pathways due to being one of the main methyl donors in metabolism [4–7]. Before methionine can function as a methyl donor, methionine needs to be converted to SAM by the enzyme S-adenosyl methyltransferase (Figure 1(a)). Upon donating the methyl group, SAM is converted to S-adenosylhomocysteine (SAH) which is unstable and easily converted to homocysteine. Homocysteine is cytotoxic to the cells and needs either to be remethylated via methionine synthase requiring folate as the methyl donor or betaine homocysteine methyltransferase using betaine (also known as trimethylglycine) as the methyl donor or eliminated via the transsulfuration pathway (Figure 1(a); [7–9]). Therefore, the SAM to SAH ratio has been used as a marker for the methylation capacity of cells [10, 11]. When dietary methionine was increased, so did SAM, indicative of increased capacity of methylation in both liver and muscle tissues of Atlantic salmon [1, 12]. The liver is a key target organ for methylation reactions where SAM is the methyl donor (Figure 1(a)) and participates in posttranslational protein modifications, DNA and histone tail methylation, as well as many metabolic pathways such as creatine synthesis [13–15], carnitine synthesis [16, 17], and endogenous phosphocholine synthesis [18]. When SAM is decarboxylated, the resulting aminopropyl group is used in polyamine synthesis (Figure 2(a), [19, 20]). Dietary methionine determines the amount of cysteine being produced as excess methionine is eliminated through either transsulfuration [1, 21, 22] or through entering the tricarboxylic acid cycle (TCA cycle) as succinyl-coenzyme A (succinyl-CoA) being either used for energy production or stored as lipids (Figure 2(b)).

## 3. Transsulfuration, Glutathione, and Taurine Synthesis

Methionine is a precursor for cysteine, one of three constituting amino acids of glutathione (GSH). GSH is a tripeptide consisting of cysteine, glutamine, and glycine. GSH is synthetized *in vivo* with two ATP-dependent steps where *γ*-glutamylcysteine is synthetized by the rate limiting enzyme glutamate–cysteine synthase. Next, the dipeptide reacts with L-glycine by the enzyme glutathione synthase (Figure 1(a), [23]). Most GSH is present in the cytosol, which also is the main place for its synthesis [24]. GSH is redistributed to cell organelles after being synthetized. The main organ synthetizing GSH is the liver. The GSH/GSSG system is important to scavenge reactive oxygen species (ROS) and oxidative stress within the cells (Figure 1(a)). Both an excess and a deficiency in methionine supply can have a significant impact on the oxidation status, as reflected by the GSH/GSSG ratio (Figure 1(a); [9]). Moreover, it can exert an allosteric influence on protein activation [25].

Taurine is synthetized from methionine through the transsulfuration of homocysteine (Figure 1(a)). Cystathionine produced through transsulfuration is transformed to cysteine of which is oxidized to cysteine sulfinate by the rate limiting enzyme of taurine synthesis cysteine dioxygenase (CDO). Cysteine sulfinate is then decarboxylated to hypotaurine by the enzyme cysteine sulfinate decarboxylase and the hypotaurine produced is spontaneously oxidized to taurine (reviewed by Parsberg Werge et al. [22]). For vertebrates, excess taurine is excreted in the urine. When dietary methionine is increased in salmon diets the level of taurine in liver, plasma, and muscle increases, indicating that salmon effectively synthetize taurine from methionine through the transsulfuration pathway [1, 21, 26]. As taurine is not present in a plant-based diet, taurine availability has been reduced when changing from a fish meal to a plant-based diet (reviewed by Salze and Davis [27]). Thus, if taurine is not added to a plant-based diet, a higher requirement of methionine and or cysteine may be necessary to provide taurine for the metabolism.

## 4. Methionine and Polyamines

Polyamines are synthetized from ornithine (produced from arginine) and decarboxylated S-adenosylmethionine (dcSAM) which is synthetized from methionine through decarboxylation of SAM. The rate limiting enzymes for polyamine synthesis are SAM decarboxylase and ornithine decarboxylase (Figure 2(a)). The polyamines spermidine and spermine are the only polyamines being synthetized in animal metabolism [19]. Polyamines are low-weight molecules that have a positive charge at physiological pH. They stabilize chromatin and nuclear enzymes forming complexes with negatively charged molecules such as DNA, RNA phospholipids, and proteins. Spermine is present in millimolar concentration within the nucleus of cells and scavenges free radicals protecting the nuclear DNA [28]. In the liver of Atlantic salmon, spermidine and spermine concentration lays in the range of 20–30 *µ*mol/100 g and 80–90 *µ*mol/100 g, respectively [2]. Polyamine concentrations within mammalian cells are regulated by the enzyme spermidine/spermine-N1-acetyltransferase (SSAT) of which acetylates polyamines so they can be metabolized to lower polyamines or being oxidized [19, 29, 30]. Any elevated SSAT activity might activate polyamine oxidases (spermidine oxidase (SMO)), acetylated polyamine oxidase (APAO), and polyamine oxidase (PAO) and produce H_2_O_2_ and aldehydes that might affect oxidation status within cells [17, 30–32]. Depletion of polyamines decreases cellular proliferation and growth ([30], Figure 1(b)). Liver spermine concentration in Atlantic salmon was more affected by the dietary methionine [2] than dietary arginine [33, 34]. Espe et al. [35] reported that increased spermine increased the gene expression of SSAT in primary liver cells isolated from Atlantic salmon, indicating that salmon also regulates polyamine concentrations within the cells through SSAT.

## 5. Methionine and Synthesis of Carnitine and Creatine

Creatine is synthetized in two steps, catalyzed by L-arginine glycine amidinotransferase and guanidinoacetate *N*-methyltransferase in kidney and liver, respectively [36]. Carnitine is synthetized from peptide bound trimethyllysine, that after being hydrolyzed, are transformed to butyrobetaine and transported to the liver for hydroxylation to carnitine [16]. Carnitine and creatine are low in vegan diets as compared to diets containing meat or fish [14, 15, 37]. The change from a fish meal-based diet in salmonids to plant-based diets will then reduce the dietary carnitine and creatine if not supplemented or synthetized endogenously. The synthesis of carnitine and creatine both require SAM arriving from methionine (Figure 1(a)). Carnitine is required to transport long chain fatty acids across the mitochondrion membranes and enable *β*-oxidation and energy production, while creatine is a short-lived energy metabolite after being phosphorylated and stored in tissues to provide energy for the flight and fight response when ATP synthesis is less than the required ATP [17]. The creatinine produced upon transfer of phosphorous to ADP, is excreted in the urine. Thus, the continuous synthesis of creatine or a dietary supply is necessary to keep the creatine concentration within tissues sufficient [15]. Piglets effectively synthesize carnitine from guanidinoacetate (GAA) provided the available methionine is sufficient to support the methylation of GAA [38].

## 6. Methionine and Endogenous Synthesis of Phosphatidylcholine

Most of the phosphatidylcholine (PC) synthesis is through the Kennedy pathway, but some also might be synthetized from phosphatidylethanolamine (PEA) in the liver tissues (Figure 1(a)). The endogenous synthesis of PC from PEA requires SAM arriving from methionine through the enzyme phosphatidylethanolamine N-methyltransferase (PEMT) [18, 39–41]. PC is the main phospholipid present in the very low-density lipoprotein (VLDL) transporting lipids from the liver to peripheral tissues for energy supply or storage of energy [39]. When salmon are fed low-methionine diets the PC is reduced indicative of a reduced PEMT activity and/or a reduced availability of SAM [2]. Supplementation of choline, the precursor of betaine resulted in an increased PC concentration in liver and muscle of Atlantic salmon [2, 42].

## 7. Methionine and Epigenetics

DNA methylation and methylation of histone tails depend on the availability of methionine and SAM (Figure 1(a) and 1(b)). Both are epigenetic gene regulatory mechanisms that adjust the availability for gene transcription, but this type of gene regulation depends on the location in the genome (reviewed by Skjærven et al. [7]). Another epigenetic key regulator is acetyl-CoA that fuels the TCA cycle, and is a product of either degradation from certain amino acids, glycolysis through pyruvate or fatty acid *β*-oxidation (Figure 2(b)). Acetyl-CoA may also be utilized as substrate for histone lysine acyltransferases and can thereby affect epigenetic regulation (Figure 1(a), [7]).

The availability of SAM through the 1C metabolism is intricately linked with the B-vitamins, cobalamin (Vitamin B12), folate (Vitamin B9), and pyridoxine (Vitamin B6) acting as cofactors or methyl donors (Figure 1(a); [6, 7, 21]). Nutritional studies performed in rainbow trout (*Oncorhynchus mykiss*) and zebrafish (*Danio rerio*) showed that DNA methylation is particularly sensitive to the dietary levels of methionine, alone or in combination with the other important micronutrients such as those nutrients above mentioned [7, 43, 44]. A study with Atlantic salmon focused on potential nutritional programing through additional levels of solely methionine, folate, Vitamin B12, and B6 before and throughout smoltification to improve health, growth, and robustness after sea water transfer. They concluded that moderate surplus levels of the four nutrients improved growth in the sea water period [21]. Extra amounts of these key nutrients adjusted a range of metabolites in liver and muscle, and particularly the SAM/SAH ratio that directly impacts the methylation potential of both DNA methylation and histone tails with possible consequences for gene expression [12, 45]. The overall metabolic state and molecular profiles of muscle showed that the dietary effect before sea water transfer was intensified at postsmolt suggesting metabolic programing, possibly epigenetic programing [12]. Nutritional programing and epigenetics in teleosts were recently reviewed by Hou and Fuiman [46] and Skjærven et al. [7].

As described for mammals, the parental nutrient status of micronutrients crucial to the 1C metabolism have an uttermost importance for the health and phenotype of the next generation [47]. For fish, parental micronutrient deficiency including the Vitamins B12, B9, B6, and methionine results in an altered gene expression profile at the organogenesis stage [48], which is an evolutionary conserved developmental stage among vertebrates. Changing the parental feed also affected the embryonic gene expression of immune-, lipid transport-, and apolipoprotein genes [48], which leads to a domino effect of subsequent altered events through development. The parental feed did not change the growth of the offspring when feeding them diets with enough micronutrients. However, when the offspring reached maturity, they developed a fatty liver like phenotype, altered gene expression of the genes encoding enzymes of the cholesterol biosynthesis pathway and mitochondrial ribosomal proteins [48]. In addition, when analyzing the epigenetic profiles of their DNA we found a massive change, which suggested that 1,800 locus specific sites had altered DNA methylation due to the parental feed. Histology analysis identified a higher inclusion of lipids in the hepatocytes in offspring from the deficient parental feeding group. Recently, feeding rainbow trout (*Oncorhynchus mykiss*) with brood stock diets enriched with methionine and choline improved the growth in the offspring probably due to improvements in 1C metabolism [49], although neither DNA methylation nor 1C metabolites were analyzed.

## 8. Methionine and Effects on Lipid Metabolism and the Development of Fatty Liver

Dietary methionine levels broadly influence lipid metabolism through interactions with phosphocholine, creatine, carnitine, polyamine, and cholesterol metabolism (Figures 1 and 2), and any deficiency in methionine and/or its metabolites affects lipid accumulation.

We have earlier shown that adult salmon fed deficient methionine increased triacylglycerol (TAG) accumulation in liver [26]. Juvenile salmon, fed a low-methionine diet, did not increase liver TAG, but had an increased relative liver weight, indicating a change in liver metabolism maybe due to increased lipid storage [2, 21]. This indicates that the methionine requirement for a healthy metabolism is higher than the methionine required for growth and the requirement is higher for juveniles than an adult salmon. The more methionine salmon are fed the higher is the concentration of taurine in liver, plasma, and muscle indicating that salmon increased transsulfuration when methionine is in excess [1, 21, 26]. Taurine is also linked to oxidation as its supplementation increased antioxidant enzyme activity and decreased malondialdehyde in broilers [50] and inhibited the fatty liver development in rats [51] and mice [52]. Taurine is a component of bile acid, as taurocholic acid, and a taurine deficiency reduces the absorption of lipids in Atlantic salmon [35]. Espe et al. [53] reported a reduction in viscera mass when taurine was added to the salmon diet. This was also the case when salmon were fed fish protein hydrolysates, which is a protein source rich in taurine, resulting in reduced relative liver size and reduced visceral fat mass [54]. Also, a deficiency in methionine and choline contributes to TAG accumulation in liver of both mammals and fish as less TAG is exported as VLDL [55–57]. This may be partly due to a lack of methyl groups linked to a reduced phosphocholine status and thus reduced fat transport from the liver to peripheral tissues that may contribute to increased TAG accumulation in the liver.

Methionine, or rather the taurine synthetized from methionine through transsulfuration, affects cholesterol metabolism (Figure 1(b)) in mammals [58] and fish [43, 59]. Taurine is a component of bile acid, as taurocholic acid, and a taurine deficiency reduces the absorption of lipids [35]. Taurine administration to animal models of nonalcoholic fatty liver disease (NAFLD) decreased steatosis, inflammation, and oxidative stress (Figure 1(b); [22, 60–63]). Fish hydrolyzed proteins rich in taurine and glycine increased cholesterol excretion through increased bile acid secretion in feces and reduced mesenteric fat accumulation in rats [64]. Also, feces of Atlantic salmon contained higher bile acid content when fed the higher methionine diet as compared to fish fed lower methionine diets. Concomitantly, those fish fed the higher methionine diet contained higher liver taurine concentration [26]. Probably, taurine has a cholesterol lowering effect on cholesterol 7A-hydroxylase (CYP7A1), the rate limiting enzyme for cholesterol degradation into bile acids (reviewed by Chen et al. [58]).

Increased polyamine turnover consumes ATP and acetyl-CoA, this affects the energy sensing and lipid metabolism through increased fatty acid oxidation and thus has the potential to reduce adiposity in mammals [65, 66]. Spermine supplementation to obese mice reduced body weight, increased fatty acid oxidation as well as improved glucose utilization [67]. Thus, polyamines might have both anti-inflammatory and anti-oxidative effects within cells depending on the cell type and organelle and they are helpful for the cell to constitute the cellular structure and integrity depending on their concentration. To the best of our knowledge, polyamine supplementation and its effect on lipid storage have not been tested in the Atlantic salmon.

Both creatine and carnitine supplementation improved fatty liver disease (reviewed by Barcelos et al. [68] and Li and Zhao [69], respectively). Atlantic salmon fed carnitine supplemented diets reduced body fat and increased body protein [70]. Likewise, carnitine supplementation to high-fat diets decreased fat deposition and liver damage in largemouth bass (*Micropterus salmoides*; [71]). Carnitine supplementation in diets fed to hybrid tilapia (*Oreochromis niloticus* ^*∗*^*Oreochromis aureus*) reduced mesenteric fat without affecting growth performance or liver fat [72]. Lin et al. [58] reported that creatine supplementation to spotted sea bass (*Lateolabrax maculatus*) improved growth and muscle quality. Similar results were reported by Burns and Gatlin [73] and Schrama et al. [74] in red drum (*Sciaenops ocellatus*) and European sea bass (*Dicentrarchus labrax*), respectively. To the best of our knowledge, dietary creatine supplementation and its effect on adiposity or liver fat accumulation have not been studied in the Atlantic salmon.

## 9. Sulfur Amino Acids and Oxidation

Oxidative stress increases the methionine flux through transsulfuration resulting in increased GSH production [22, 75]. An increase in ROS will interact with the mitochondrial energy production and influence inflammation as sulfur amino acids counteract ROS as described for mammalian species (reviewed by Baliou et al. [76]; Figure 1(a) and 1(b)). Any imbalance between oxidative stress and scavenging with anti-oxidation may increase ROS and disturb the cells and tissues. The enzymes catalase, glutathione peroxidase (GPX) and superoxide dismutase (SOD) are the most important antioxidative enzymes protecting tissues against oxidative stress scavenging the oxide radicals and hydrogen peroxide. In addition, cells have the glutathione (GSH) system scavenging H_2_O_2_ by being oxidized to GSSG. GSSG is reduced back by NADPH-dependent GSH reductase and the thioredoxin system (Figure 1(a)). The synthesis of glutathione occurs in cytosol [24]. Cysteine availability is the rate limiting of the three amino acids constituting GSH and is available in low concentrations in cells [23, 77]. The concentration of L-cysteine is approximately 10 *µ*mol/100 g in the liver and below detectable levels in the muscle of Atlantic salmon [1]. Cysteine arrives from either feed consumption, protein degradation or through transsulfuration of homocysteine produced in the 1C metabolism (Figure 1(a); [21, 78]). Mitochondrial DNA does not contain protective histone proteins and has a low-DNA repair activity leaving it more vulnerable to ROS than is the nuclear DNA [79]. Cysteine released following GSH degradation is unstable and not reused, and any increased turnover of glutathione requires a steady supply of cysteine [80]. As cysteine is very unstable in the biological tissues, N-acetylcysteine (NAC) often is added to diets, being a stable cysteine component and nontoxic to animals, thus sparing methionine and SAM for other metabolic pathways ([80] and references therein). Rainbow trout fed methionine deficient diets are reported to have mitochondrial defects and decreased oxidative status (GSH : GSSG ratio; [81]). Grass carp (*Ctenopharyngodon idella*) fed diets containing NAC reduced oxidation by increasing the synthesis of glutathione [82]. Atlantic salmon fed diets added glutathione improved oxidative status, but when doses exceeded about 200 mg/kg the oxidation increased [83]. Similar results were also reported in rainbow trout intestine post being fed glutathione [56].

## 10. Methionine and Inflammation

ROS is tightly linked to inflammation, and a methionine–choline-deficient diet increased both steatosis and inflammation in a rodent model [55]. Taurine is also linked to ROS and present in high concentrations in organs producing the ROS, although its exact mechanisms are not well known (reviewed by Marcinkiewicz and Kontny [84]). The methionine–choline-deficient diet resulted in increased pro-inflammatory markers (IL-1b, tumor necrosis factor alpha (TNF*α*), and IL-6), increased ROS as well as increased lipid accumulation in mice [85]. Concomitantly, most of the antioxidative enzymes were reduced (SOD, GPX, and catalase) in those mice fed the methionine–choline-deficient diet. In salmon, inflammation following deficiency and abundance of sulfur amino acids have not been studied through a low-methionine diet that increased gene expression of TNF*α* and reduced the expression of GPX-3 as compared to salmon fed adequate methionine diets [2]. Recently, Zhang et al. [86] reported reduced inflammation when juvenile turbot (*Scophthalmus maximus* L.) was fed taurine following a high carbohydrate induced stress. Machado et al. [87] reported improved immune response in European sea bass (*Dicentrarchus labrax*) fed methionine enriched diets. Also, any imbalances in polyamines have been linked to inflammation, as the activity of SSAT increased during inflammation [88]. To the best of our knowledge, polyamines and inflammation have not been studied in the Atlantic salmon. Future fish nutrition studies are required to link sulfur amino acids to inflammation including the interaction with the oxidation status in the Atlantic salmon (reviewed by Wang et al. [3]; Figure 1(b)).

## 11. Concluding Remarks

As methionine is an important precursor for many metabolites and methylation reactions, it is apparent that the availability of dietary methionine has consequences for metabolism, and possibly also disease states such as adiposity and the development of fatty liver. During the last decades salmon diets have changed from animal origin, containing relatively high amounts of taurine, carnitine, and creatine, to plant-based diets containing none or very low levels of these metabolites, which results in a need for endogenous synthesis and competition for the methyl donor SAM. The main plant protein ingredient used in salmon feed is soy protein, which is low in methionine. This emphasizes that the methionine requirement for healthy metabolism is higher than for the actual growth. We believe that the future will increase our knowledge of the interactions between methionine availability and metabolic pathways being the most important to secure a healthy metabolism of Atlantic salmon. Profound research on recommendations for methionine, sulfur amino acids, and metabolites that require methyl donors for their endogenous synthesis, will increase knowledge on the metabolites that might be added to the future salmon diets to improve metabolic and overall health and welfare of the farmed salmon.

## Data Availability

This is a review and data used are in the reference list.

## References

[1] Espe M., Hevrøy E. M., Liaset B., Lemme A., El-Mowafi A. (2008). Methionine intake affect hepatic sulphur metabolism in Atlantic salmon, *Salmo salar*. *Aquaculture*.

[2] Espe M., Andersen S. M., Holen E. (2014). Methionine does not increase polyamine turnover through depletion of hepatic S-adenosylmethionine in juvenile Atlantic salmon. *British Journal of Nutrition*.

[3] Wang L., Gao C., Wang B., Wang C., Sagada G., Yan Y. (2023). Methionine in fish health and nutrition: potential mechanisms, affecting factors, and future perspectives. *Aquaculture*.

[4] Finkelstein J. D. (1990). Methionine metabolism in mammals. *The Journal of Nutritional Biochemistry*.

[5] Mato J. M., Corrales F. J., Lu S. C., Avila M. A. (2002). S-adenosylmethionine: a control switch that regulates liver function. *The FASEB Journal*.

[6] Ducker G. S., Rabinowitz J. D. (2017). One-carbon metabolism in health and disease. *Cell Metabolism*.

[7] Skjærven K. H., Adam A.-C., Takaya S., Waagbø R., Espe M., Fernandez I., Fernandez J. (2022). Nutritional epigenetics. *Cellular and Molecular Approaches in Fish Biology*.

[8] Obeid R. (2013). The metabolic burden of methyl donor deficiency with focus on the betaine homocysteine methyltransferase pathway. *Nutrients*.

[9] Aissa A. F., Tryndyak V., deConti A. (2014). Effect of methionine-deficient and methionine-supplemented diets on the hepatic one-carbon and lipid metabolism in mice. *Molecular Nutrition & Food Research*.

[10] Kerr S. J. (1972). Competing methyltransferase systems. *Journal of Biological Chemistry*.

[11] Cantoni G. L., Chiang P. K., Cavallini D., Gaull G. E., Zappia V. (1980). The role of S-adenosylhomocysteine hydrolase in the control of biological methylations. *Natural Sulphur Compounds*.

[12] Adam A.-C., Saito T., Espe M. (2022). Metabolic and molecular signatures of improved growth in Atlantic salmon (*Salmo salar*) fed surplus levels of methionine, folic acid, vitamin B_6_ and B_12_ throughout smoltification. *British Journal of Nutrition*.

[13] Brosnan J. T., Jacobs R. L., Stead L. M., Brosnan M. E. (2004). Methylation demand: a key determinant of homocysteine metabolism. *Acta Biochimica Polonica*.

[14] Brosnan J. T., da Silva R. P., Brosnan M. E. (2011). The metabolic burden of creatine synthesis. *Amino Acids*.

[15] Brosnan M. E., Brosnan J. T. (2016). The role of dietary creatine. *Amino Acids*.

[16] Arslan C. (2006). L-carnitine and its use as a feed additive in poultry feeding a review. *Revue de Medicine Veterinaire*.

[17] Cecero R. A., Pegg A. (2009). Polyamine catabolism and disease. *Biochemical Journal*.

[18] Noga A. A., Vance D. E. (2003). Insights into the requirement of phosphatidylcholine synthesis for liver function in mice. *Journal of Lipid Research*.

[19] Pegg A. E. (2009). Mammalian polyamine metabolism and function. *IUBMB Life*.

[20] da Silva R. P., Nissim I., Brosnan M. E., Brosnan J. T. (2009). Creatine synthesis: hepatic metabolism of guanidinoacetate and creatine in the rat in vitro and in vivo. *American Journal of Physiology-Endocrinology and Metabolism*.

[21] Espe M., Vikeså V., Helgøy Thomsen T., Adam A.-C., Saito T., Skjærven K. H. (2020). Atlantic salmon fed a nutrient package of surplus methionine, vitamin B12, folic acid and vitamin B6 improved growth and reduced the relative liver size, but when in excess growth reduced. *Aquaculture Nutrition*.

[22] Werge M. P., McCann A., Galsgaard E. D. (2021). The role of transsulfuration pathway in non-alcoholic fatty liver disease. *Journal of Clinical Medicine*.

[23] Lu S. J. (2013). Glutathione synthesis. *Biochimica et Biophysica Acta (BBA) - General Subjects*.

[24] Wu G., Fang Y.-Z., Yang S., Lupton J. R., Turner N. D. (2004). Glutathione metabolism and its implication for health. *Journal of Nutrition*.

[25] Aledo J. C. (2019). Methionine in proteins: the Cinderella of the proteinogenic amino acids. *Protein Science*.

[26] Espe M., Rathore R. M., Du Z.-Y., Liaset B., El-Mowafi A. (2010). Methionine limitation results in increased hepatic FAS activity, higher liver 18:1 to 18:0 fatty acid ratio and hepatic TAG accumulation in Atlantic salmon, *Salmo salar*. *Amino Acids*.

[27] Salze G. P., Davis D. A. (2015). Taurine: a critical nutrient for future fish feeds. *Aquaculture*.

[28] Ha H. C., Sirisoma N. S., Kuppusamy P., Zweier J. L., Woster P. M., Casero R. A. (1998). The natural polyamine spermine functions directly as a free radical scavenger. *Proceedings of the National Academy of Sciences*.

[29] Agostinelli E., Arancia G., Vedova L. D. (2004). The biological functions of polyamine oxidation products by amine oxidases: perspectives and clinical applications. *Amino Acids*.

[30] Pegg A. E. (2008). Spermidine/spermine- *N*^1^ -acetyltransferase: a key metabolic regulator. *American Journal of Physiology-Endocrinology and Metabolism*.

[31] Cervelli M., Amendola R., Polticelli F., Mariottini P. (2012). Spermine oxidase: ten years after. *Amino Acids*.

[32] Babbar N., Murray-Stewart T., Casero R. A. (2007). Inflammation and polyamine catabolism: the good, the bad and the ugly. *Biochemical Society Transactions*.

[33] Andersen S. M., Holen E., Aksnes A., Rønnestad I., Zerrahn J.-E., Espe M. (2013). Dietary arginine affects energy metabolism through polyamine turnover in juvenile Atlantic salmon (*Salmo salar*). *British Journal of Nutrition*.

[34] Andersen S. M., Holen E., Aksnes A., Rønnestad I., Zerrahn J.-E., Espe M. (2015). Adult Atlantic salmon (*Salmo salar* L.) adapts to high levels of dietary arginine supplementation, by increasing metabolism of arginine and polyamine turnover in liver. *Aquaculture Nutrition*.

[35] Espe M., Liaset B., Hevrøy E. M., El-Mowafi A. (2011). DL-methionine enrichment in diets fed to Atlantic salmon increases apparent digestibility. *Aquaculture Research*.

[36] Wuertz S., Reiser S. (2023). Creatine: a valuable supplement in aquafeeds?. *Reviews in Aquaculture*.

[37] Wu G. (2020). Important roles of dietary taurine, creatine, carnosine, anserine and 4-hydroxyproline in human nutrition and health. *Amino Acids*.

[38] Dinesh O. C., Kankayaliyan T., Rademacher M., Tomlinson C., Bertolo R. F., Brunton J. A. (2021). Neonatal piglets can synthesize adequate creatine, but only with sufficient dietary arginine and methionine, or with guanidinoacetate and excess methionine. *The Journal of Nutrition*.

[39] van der Veen J. N., Kennelly J. P., Wan S., Vance J. E., Vance D. E., Jacobs R. L. (2017). The critical role of phosphatidylcholine and phosphatidylethanolamine metabolism in health and disease. *Biochimica et Biophysica Acta (BBA) - Biomembranes*.

[40] Gibellini F., Smith T. K. (2010). The Kennedy pathway—de novo synthesis of phospatidylethanolamine and phosphatidylcholine. *IUMB Life*.

[41] Cui Z., Vance D. E. (1996). Expression of phosphatidylethanolamine N-methyltransferase-2 is markedly enhanced in long term choline-deficient rats.. *The Journal of Biological Chemistry*.

[42] Espe M., Zerrahn J.-E., Holen E., Rønnestad I., Veiseth-Kent E., Aksnes A. (2016). Choline supplementation to low methionine diets increase phospholipids in Atlantic salmon, while taurine supplementation had no effects on phospholipid status, but improved taurine status. *Aquaculture Nutrition*.

[43] Skjærven K. H., Jakt L. M., Fernandes J. M. O. (2018). Parental micronutrient deficiency distorts liver DNA methylation and expression of lipid genes associated with a fatty-liver-like phenotype in offspring. *Scientific Reports*.

[44] Veron V., Marandel L., Liu J. (2018). DNA methylation of the promoter region of bnip3 and bnip3l genes induced by metabolic programming. *BMC Genomics*.

[45] Saito T., Espe M., Vikeså V. (2023). One-carbon metabolism nutrients impact the interplay between DNA methylation and gene expression in liver, enhancing protein synthesis in Atlantic Salmon. *bioRxiv*.

[46] Hou Z. X., Fuiman L. A. (2020). Nutritional programming in fishes: insight from mammalian studies. *Reviews in Fish Biology and Fisheries*.

[47] Korsmo H. W., Jiang X. (2021). One carbon metabolism and early development: a diet-dependent destiny. *Trends in Endocrinology & Metabolism*.

[48] Skjærven K. H., Jakt L. M., Dahl J. A. (2016). Parental vitamin deficiency affects the embryonic gene expression of immune-, lipid transport- and apolipoprotein genes. *Scientific Reports*.

[49] Cleveland B. M., Leeds T. D., Picklo M. J., Brentesen C., Frost J., Biga P. R. (2020). Supplementing rainbow trout (*Oncorhynchus mykiss*) broodstock diets with choline and methionine improves growth in offspring. *Journal of the World Aquaculture Society*.

[50] Han H. L., Zhang J. F., Yan E. F. (2020). Effects of taurine on growth performance, antioxidant capacity, and lipid metabolism in broiler chickens. *Poultry Science*.

[51] Song Q., Guo J., Zhang Y., Chen W. (2021). The beneficial effects of taurine in alleviating fatty liver disease. *Journal of Functional Foods*.

[52] Kim K. S., Jang M. J., Fang S. (2019). Anti-obesity effect of taurine through inhibition of adipogenesis in white fat tissue but not in brown fat tissue in a high-fat diet-induced obese mouse model. *Amino Acids*.

[53] Espe M., Ruohonen K., El-Mowafi A. (2012a). Effect of taurine supplementation on metabolism and body lipid to protein ratio in juvenile Atlantic salmon (*Salmo salar*). *Aquaculture Research*.

[54] Espe M., Ruohonen K., El-Mowafi A. (2012). Hydrolysed fish protein concentrate (FPC) reduces viscera mass in Atlantic salmon (Salmo salar) fed plant-protein-based diets. *Aquaculture Nutrition*.

[55] Wang X., Hausding M., Weng S.-Y. (2018). Gliptins suppress inflammatory macrophage activation to mitigate inflammation, fibrosis, oxidative stress, and vascular dysfunction in models of nonalcoholic steatohepatitis and liver fibrosis. *Antioxidants & Redox Signaling*.

[56] Wang C., Su B., Lu S. (2021). Effects of glutathione on growth, intestinal antioxidant capacity, histology, gene expression and microbiota of juvenile triploid *Oncorhynchus mykiss*. *Frontiers in Physiology*.

[57] Bai F., Niu X., Wang X., Ye J. (2021). Growth performance, biochemical composition and expression of lipid metabolism related genes in groupers (*Epinephelus coioides*) are altered by dietary taurine. *Aquaculture Nutrition*.

[58] Chen W., Guo J.-X., Chang P. (2012). The effect of taurine on cholesterol metabolism. *Molecular Nutrition & Food Research*.

[59] Saito T., Whatmore P., Taylor J. F. (2021). Micronutrient supplementation affects transcriptional and epigenetic regulation of lipid metabolism in a dose-dependent manner. *Epigenetics*.

[60] Murakami S., Ono A., Kawasaki A., Takenaga T., Ito T. (2018). Taurine attenuates the development of hepatic steatosis through the inhibition of oxidative stress in a model of nonalcoholic fatty liver disease in vivo and in vitro. *Amino Acids*.

[61] Murakami S. (2015). Role of taurine in the pathogenesis of obesity. *Molecular Nutrition & Food Research*.

[62] Gentile C. L., Nivala A. M., Gonzales J. C. (2011). Experimental evidence for therapeutic potential of taurine in the treatment of nonalcoholic fatty liver disease. *American Journal of Physiology-Regulatory, Integrative and Comparative Physiology*.

[63] Dong Y., Li X., Liu Y., Gao J., Tao J. (2021). The molecular targets of taurine confer anti-hyperlipidemic effects. *Life Sciences*.

[64] Liaset B., Madsen L., Hao Q. (2009). Fish protein hydrolysate elevates plasma bile acids and reduces visceral adipose tissue mass in rats. *Biochimica et Biophysica Acta (BBA) - Molecular and Cell Biology of Lipids*.

[65] Jell J., Merali S., Hensen M. L. (2007). Genetically altered expression of spermidine/spermine N1-acetyltransferase affects fat metabolism in mice via acetyl-CoA.. *The Journal of Biological Chemistry*.

[66] Pirinen E., Kuulasmaa T., Pietilä M. (2007). Enhanced polyamine catabolism alters homeostatic control of white adipose tissue mass, energy expenditure, and glucose metabolism.. *Molecular and Cellular Biology*.

[67] Sadasivan S. K., Vasamsetti B., Singh J. (2014). Exogenous administration of spermine improves glucose utilization and decreases body weight in mice. *Endocrine Pharmacology*.

[68] Barcelos R. P., Stefanello S. T., Mauriz J. L., Gonzalez-Gallego J., Soares F. A. A. (2016). Creatine and the liver: metabolism and possible interactions. *Mini-Reviews in Medicinal Chemistry*.

[69] Li N., Zhao H. (2021). Role of carnitine in non-alcoholic fatty liver disease and other related diseases: an update. *Frontiers in Medicine*.

[70] Ji H., Bradley T. M., Tremblay G. C. (1996). Atlantic salmon (*Salmo salar*) fed L-carnitine exhibit altered intermediary metabolism and reduced tissue lipid, but no change in growth rate.. *The Journal of Nutrition*.

[71] Liu Y.-C., Limbu S. M., Wang J.-G. (2022). Dietary L-carnitine alleviates the adverse effects caused by reducing protein and increasing fat contents in diet juvenile largemouth bass (*Micropterus salmoides*). *Aquaculture Nutrition*.

[72] Yang S.-D., Wen Y.-C., Liou C.-H., Liu F.-G. (2009). Influence of dietary L-carnitine on growth, biological traits and meat quality in tilapia. *Aquaculture Research*.

[73] Burns A. F., Gatlin D. M. (2019). Dietary creatine requirement of red drum (*Sciaenops ocellatus*) and effects of water salinity on response to creatine supplementation. *Aquaculture*.

[74] Schrama D., deMagalhaes R., Cerqueira M. (2022). Effect of creatine and EDTA supplemented diets on European seabass (*Dicentrarchus labrax*) allergenicity, fish muscle quality and omics fingerprint. *Comparative Biochemistry and Physiology Part D*.

[75] Vivitsky V., Mosharov E., Tritt M., Ataullakhanov F., Banerjee R. (2003). Redox regulation of homocysteine-dependent glutathione synthesis. *Redox Report*.

[76] Baliou S., Adamaki M., Ioannou P. (2021). Protective role of taurine against oxidative stress (review). *Molecular Medicine Reports*.

[77] Anderson M. E., Meister A. (1983). Transport and direct utilization of gamma-glutamylcyst(e)ine for glutathione synthesis.. *Proceedings of the National Academy of Sciences*.

[78] Li P., Mai K., Trushenski J., Wu G. (2009). New developments in fish amino acid nutrition: towards functional and environmentally oriented aquafeeds. *Amino Acids*.

[79] Cardenas E., Davies K. J. (2000). Mitochondrial free radical generation, oxidative stress, and aging. *Free Radical Biology and Medicine*.

[80] Morris G., Anderson G., Dean O. (2014). The glutathione system: a new drug target in neuroimmune disorders. *Molecular Neurobiology*.

[81] Séité S., Mourier A., Camougrand N. (2018). Dietary methionine deficiency affects oxidative status, mitochondrial integrity and mitophagy in the liver of rainbow trout (*Oncorhynchus mykiss*). *Scientific Reports*.

[82] Xie S., Tian L., Niu J., Liang G., Liu Y. (2017). Effect of N-acetyl cysteine and glycine supplementation on growth performance, glutathione synthesis, and antioxidative ability of grass carp, *Ctenopharyngodon idella*. *Fish Physiology and Biochemistry*.

[83] Ma J., Zhang J., Sun G., Lou Y., Li Y. (2019). Effects of dietary reduced glutathione on the growth and antioxidative capacity of juvenile Atlantic salmon (*Salmo salar*). *Aquaculture Nutrition*.

[84] Marcinkiewicz J., Kontny E. (2014). Taurine and inflammatory diseases. *Amino Acids*.

[85] Heo Y. J., Choi S.-E., Lee J. (2021). Visfatin exacerbates hepatic inflammation and fibrosis in a methionine–choline-deficient diet mouse model. *Hepatology*.

[86] Zhang Y., Wei Z., Yang M. (2021). Dietary taurine modulates hepatic oxidative status, ER stress and inflammation in juvenile turbot (*Scophthalmus maximus* L.) fed high carbohydrate diets. *Fish & Shellfish Immunology*.

[87] Machado M., Azeredo R., Fontinha F. (2018). Dietary methionine improves the European seabass (*Dicentrarchus labrax*) immune status, inflammatory response, and disease resistance. *Frontiers in Immunology*.

[88] Agostinelli E. (2012). Role of polyamines, their analogs and transglutaminases in biological and clinical perspectives. *Amino Acids*.

